# 1-Dimethyl­amino-9,10-anthraquinone

**DOI:** 10.1107/S1600536811006829

**Published:** 2011-02-26

**Authors:** Paweł Niedziałkowski, Joanna Narloch, Damian Trzybiński, Tadeusz Ossowski

**Affiliations:** a Faculty of Chemistry, University of Gdańsk, J. Sobieskiego 18, 80-952 Gdańsk, Poland

## Abstract

In the crystal structure of the title compound, C_16_H_13_NO_2_, adjacent mol­ecules are linked through C—H⋯π and π–π [centroid–centroid distances = 3.844 (2) Å] contacts. The anthracene ring system and dimethyl­amino group are oriented at a dihedral angle of 38.4 (1)°. In the crystal, the mean planes of adjacent anthracene units are inclined at angles of 59.3 (1), 75.7 (1) and 76.0 (1)°.

## Related literature

For general background to anthraquinones, see: Arai *et al.* (1985 ▶); Dalliya *et al.* (2007 ▶); Gatto *et al.* (1996 ▶); Kowalczyk *et al.* (2010 ▶); Mori *et al.* (1990 ▶); Ossowski *et al.* (2005 ▶); Zoń *et al.* (2003 ▶). For a related structure, see: Yatsenko *et al.* (2000 ▶). For mol­ecular inter­actions, see: Hunter *et al.* (2001 ▶); Spek (2009 ▶); Takahashi *et al.* (2001 ▶).
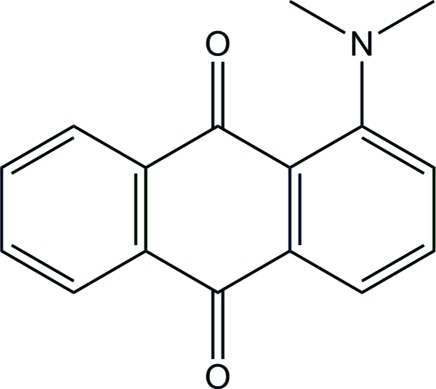


## Experimental

### 

#### Crystal data

C_16_H_13_NO_2_
*M* *_r_* = 251.27
Orthorhombic, 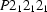

*a* = 7.2823 (3) Å
*b* = 11.1519 (7) Å
*c* = 14.9834 (7) Å
*V* = 1216.82 (11) Å^3^
*Z* = 4
Mo *K*α radiation
μ = 0.09 mm^−1^
*T* = 295 K
0.45 × 0.20 × 0.18 mm

#### Data collection

Oxford Diffraction Gemini R ULTRA Ruby CCD diffractometer
4683 measured reflections
1258 independent reflections
918 reflections with *I* > 2σ(*I*)
*R*_int_ = 0.033

#### Refinement

*R*[*F* ^2^ > 2σ(*F* ^2^)] = 0.037
*wR*(*F* ^2^) = 0.079
*S* = 0.96
1258 reflections
174 parameters
H-atom parameters constrained
Δρ_max_ = 0.12 e Å^−3^
Δρ_min_ = −0.18 e Å^−3^

### 

Data collection: *CrysAlis CCD* (Oxford Diffraction, 2008 ▶); cell refinement: *CrysAlis RED* (Oxford Diffraction, 2008 ▶); data reduction: *CrysAlis RED*; program(s) used to solve structure: *SHELXS97* (Sheldrick, 2008 ▶); program(s) used to refine structure: *SHELXL97* (Sheldrick, 2008 ▶); molecular graphics: *ORTEP-3* (Farrugia, 1997 ▶); software used to prepare material for publication: *SHELXL97* and *PLATON* (Spek, 2009 ▶).

## Supplementary Material

Crystal structure: contains datablocks global, I. DOI: 10.1107/S1600536811006829/ng5119sup1.cif

Structure factors: contains datablocks I. DOI: 10.1107/S1600536811006829/ng5119Isup2.hkl

Additional supplementary materials:  crystallographic information; 3D view; checkCIF report

## Figures and Tables

**Table 1:** Hydrogen-bond geometry (Å, °) *Cg*1 and *Cg*2 are the centroids of the C1–C4/C11/C12 and C5–C8/C13/C14 rings respectively.

*D*—H⋯*A*	*D*—H	H⋯*A*	*D*⋯*A*	*D*—H⋯*A*
C2—H2⋯*Cg*1^i^	0.93	2.99	3.724 (3)	137
C4—H4⋯*Cg*2^ii^	0.93	2.81	3.678 (3)	156

Symmetry codes: (i) 
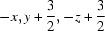
; (ii) 
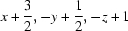
.

**Fig. 1.**
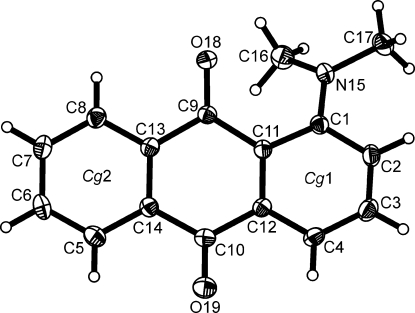
The molecular structure of the title compound showing the atom-labeling scheme. Displacement ellipsoids are drawn at the 25% probability level, and H atoms are shown as small spheres of arbitrary radius. Cg1 and Cg2 are the centroids of the C1—C4/C11/C12 and C5—C8/C13/C14 rings respectively.

**Fig. 2.**
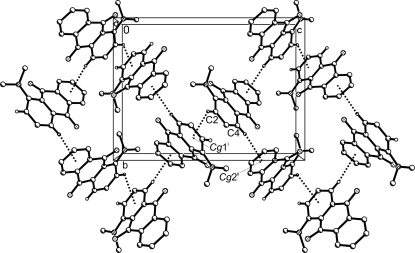
The arrangement of the molecules in the crystal structure viewed approximately along a direction. The C–H···π interactions are represented by dotted lines. H atoms not involved in interactions have been omitted. [Symmetry codes: (i) –x, y + 3/2, –z + 3/2; (ii) x + 3/2, –y + 1/2, –z + 1.]

**Fig. 3.**
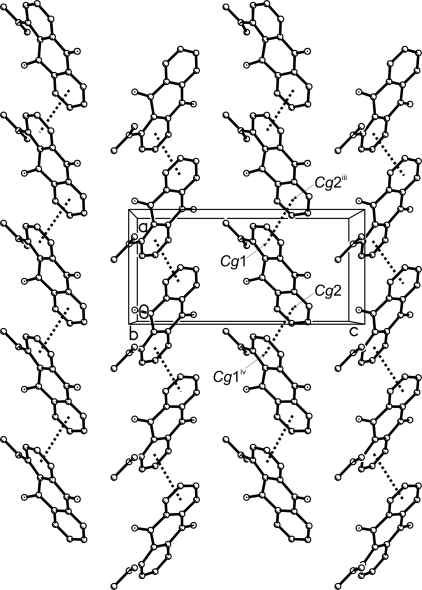
The arrangement of the molecules in the crystal structure viewed approximately along b direction. The π-π interactions are represented by dotted lines. H atoms have been omitted for clarity. [Symmetry codes: (iii) x + 1, y, z; (iv) x – 1, y + 1, z + 1.]

## supplementary crystallographic information

### Comment

Anthraquinones, the largest group of naturally occurring quinones, present in bacteria, fungi and many higher plant families contain π-electrons, reducible *p*-quinone system and are redoxactive (Zoń *et al.*, 2003). That is the reason why those found many practical applications (Kowalczyk *et al.*, 2010; Ossowski *et al.*, 2005). Both natural and synthetic derivatives have been used as colourants in food, cosmetics, textiles and hair dyes (Mori *et al.*, 1990). In medicine they are known as antitumor drugs and antibacterial or anti-inflammatory agents (Gatto *et al.*, 1996). Among anthraquinones, the amino-derivatives, due to the possibility of their chemical modification, reveal greatest potential of application. Here, we present the crystal structure of the 1-(dimethylamino)-9,10-anthraquinone – compound with interesting photophysical properties (Arai *et al.*, 1985; Dalliya *et al.*, 2007).

In the molecule of the title compound (Fig. 1), likewise in the 1-(methyl(phenyl)amino)anthraquinone (Yatsenko *et al.*, 2000), relatively strong deviation of planarity of the anthraquinone skeleton is observed. In case of the title compound, such distortion (0.1274 (3) Å) is directly caused by the steric effect of the bulky –N(CH_3_)_2_ group (Dalliya *et al.*, 2007). The dimethylamino group is twisted at an angle of 38.4 (1)° relative to the anthracene fragment. The neighboring anthracene moieties are inclined at an angle of 59.3 (1)°, 75.7 (1)° and 76.0 (1)° in the crystal lattice.

In the crystal structure, the adjacent molecules are linked by C–H···π (Table 2, Fig. 2) and π-π [centroid-centroid distances = 3.844 (2) Å] (Table 3, Fig. 3) contacts. All interactions demonstrated were found by *PLATON* (Spek, 2009). The C–H···π interactions should be of an attractive nature (Takahashi *et al.*, 2001), like the π-π (Hunter *et al.*, 2001) interactions.

### Experimental

1-(Dimethylamino)-9,10-anthraquinone was synthesized according to the procedure described below. The solution of 40% dimethylamine in water (2,21 ml, 12.36 mmol) was added to 1-chloro-9,10-anthraquinone (1 g, 4.12 mmol) in 15 ml toluene. The mixture was stirred at 130° for 4 h. The progress of the reaction was monitored by TLC (SiO_2_, dichloromethane) until the completion of reaction. The resulting mixture was concentrated to remove the solvent and dissolved in 100 ml of dichloromethane. The solution was washed with water (100 ml), the organic phase was dried over MgSO_4_ and concentrated. The resultant solid was purified by column chromatography using dichloromethane as a solvent obtaining the title compound as a red solid (921 mg, 89%). The product was recrystallized by slow evaporation from methanol to give red crystals suitable for X-ray diffraction (m.p. 137.5–137.9°C). Spectral data: IR (KBr): 3584, 2916, 2806, 1662, 1637, 1551, 1499, 1374, 1311,1270, 1180, 1024, 935, 793, 731,704 cm^-1^; ^1^H NMR (CDCl_3_, 400 MHz): 3.03 (6*H*, s, CH_3_), 7.34–7.36 (1*H*, d, J_1_ = 8.8 Hz, H-2-Ar), 7.54–7.58 (1*H*, t, J_1_ = J_2_ = 8.0 Hz, H-3-Ar), 7.69–7.72 (1*H*, t, J_1_ = 7.6 Hz, J_1_ = 6.8 Hz, J_2_ = 7.2 Hz, H-6-Ar), 7.74–7.76 (1*H*, d, J_1_ = 7.6 Hz, H-4-Ar), 7.78–7.82 (1*H*, t, J_1_ = 7.6 Hz, J_1_ = 8.4 Hz, J_2_ = 8.0 Hz, H-7-Ar), 8.22–8.24 (1*H*, t, J_1_ = 7.2 Hz, H-8-Ar). Elemental analysis: calculated for C_16_H_13_NO_2_: C 76.48, H 5.21, N 5.57; found: C 76.52, H 5.28, N 5.51.

### Refinement

1063 Friedel pairs were merged. H atoms were positioned geometrically, with C—H = 0.93 Å and 0.96 Å for the aromatic and methyl H atoms, respectively, and constrained to ride on their parent atoms with *U*_iso_(H) = *xU*_eq_(C), where *x* = 1.2 for the aromatic and *x* = 1.5 for the methyl H atoms.

### Crystal data

**Table:**

C_16_H_13_NO_2_	*F*(000) = 528
*M**_r_* = 251.27	*D*_x_ = 1.372 Mg m^−^^3^
Orthorhombic, *P*2_1_2_1_2_1_	Mo *K*α radiation, λ = 0.71073 Å
Hall symbol: P 2ac 2ab	Cell parameters from 1944 reflections
*a* = 7.2823 (3) Å	θ = 3.1–29.0°
*b* = 11.1519 (7) Å	µ = 0.09 mm^−^^1^
*c* = 14.9834 (7) Å	*T* = 295 K
*V* = 1216.82 (11) Å^3^	Prism, red
*Z* = 4	0.45 × 0.20 × 0.18 mm

### Data collection

**Table:**

Oxford Diffraction Gemini R ULTRA Ruby CCD diffractometer	918 reflections with *I* > 2σ(*I*)
Radiation source: Enhance (Mo) X-ray Source	*R*_int_ = 0.033
graphite	θ_max_ = 25.1°, θ_min_ = 3.1°
Detector resolution: 10.4002 pixels mm^-1^	*h* = −8→6
ω scans	*k* = −12→13
4683 measured reflections	*l* = −17→13
1258 independent reflections	

### Refinement

**Table:**

Refinement on *F*^2^	Primary atom site location: structure-invariant direct methods
Least-squares matrix: full	Secondary atom site location: difference Fourier map
*R*[*F*^2^ > 2σ(*F*^2^)] = 0.037	Hydrogen site location: inferred from neighbouring sites
*wR*(*F*^2^) = 0.079	H-atom parameters constrained
*S* = 0.96	*w* = 1/[σ^2^(*F*_o_^2^) + (0.0492*P*)^2^] where *P* = (*F*_o_^2^ + 2*F*_c_^2^)/3
1258 reflections	(Δ/σ)_max_ < 0.001
174 parameters	Δρ_max_ = 0.12 e Å^−^^3^
0 restraints	Δρ_min_ = −0.18 e Å^−^^3^

### Special details

**Table:**

Geometry. All e.s.d.'s (except the e.s.d. in the dihedral angle between two l.s. planes) are estimated using the full covariance matrix. The cell e.s.d.'s are taken into account individually in the estimation of e.s.d.'s in distances, angles and torsion angles; correlations between e.s.d.'s in cell parameters are only used when they are defined by crystal symmetry. An approximate (isotropic) treatment of cell e.s.d.'s is used for estimating e.s.d.'s involving l.s. planes.

### Fractional atomic coordinates and isotropic or equivalent isotropic displacement parameters (Å^2^)

**Table:**

	*x*	*y*	*z*	*U*_iso_*/*U*_eq_	
C1	0.7457 (3)	0.5675 (2)	0.53415 (14)	0.0349 (6)	
C2	0.8768 (3)	0.6609 (3)	0.53396 (17)	0.0437 (7)	
H2	0.9738	0.6578	0.4937	0.052*	
C3	0.8647 (4)	0.7554 (3)	0.59116 (18)	0.0486 (7)	
H3	0.9530	0.8155	0.5892	0.058*	
C4	0.7235 (3)	0.7630 (2)	0.65179 (17)	0.0422 (6)	
H4	0.7206	0.8255	0.6928	0.051*	
C5	0.1522 (4)	0.6067 (3)	0.78267 (16)	0.0459 (7)	
H5	0.1608	0.6622	0.8288	0.055*	
C6	0.0081 (4)	0.5277 (3)	0.78088 (18)	0.0487 (7)	
H6	−0.0795	0.5290	0.8260	0.058*	
C7	−0.0067 (3)	0.4466 (3)	0.71212 (17)	0.0465 (7)	
H7	−0.1065	0.3946	0.7099	0.056*	
C8	0.1262 (3)	0.4422 (2)	0.64640 (17)	0.0409 (6)	
H8	0.1164	0.3863	0.6006	0.049*	
C9	0.4157 (3)	0.5148 (2)	0.57716 (15)	0.0334 (6)	
C10	0.4341 (4)	0.6937 (2)	0.71603 (16)	0.0394 (7)	
C11	0.5878 (3)	0.5814 (2)	0.58994 (14)	0.0325 (6)	
C12	0.5869 (3)	0.6781 (2)	0.65160 (16)	0.0335 (6)	
C13	0.2744 (3)	0.5206 (2)	0.64829 (15)	0.0335 (6)	
C14	0.2856 (3)	0.6048 (2)	0.71661 (14)	0.0354 (6)	
N15	0.7757 (3)	0.4671 (2)	0.48311 (13)	0.0421 (6)	
C16	0.7173 (4)	0.3491 (2)	0.51109 (19)	0.0528 (8)	
H16A	0.6745	0.3525	0.5716	0.079*	
H16B	0.8189	0.2945	0.5072	0.079*	
H16C	0.6198	0.3219	0.4730	0.079*	
C17	0.9195 (3)	0.4667 (3)	0.41526 (17)	0.0552 (8)	
H17A	0.9068	0.5362	0.3780	0.083*	
H17B	0.9086	0.3957	0.3794	0.083*	
H17C	1.0376	0.4679	0.4437	0.083*	
O18	0.3823 (2)	0.45881 (18)	0.50827 (11)	0.0484 (5)	
O19	0.4317 (3)	0.77920 (19)	0.76763 (15)	0.0644 (6)	

### Atomic displacement parameters (Å^2^)

**Table:**

	*U*^11^	*U*^22^	*U*^33^	*U*^12^	*U*^13^	*U*^23^
C1	0.0328 (13)	0.0405 (16)	0.0315 (12)	0.0014 (14)	−0.0002 (12)	0.0029 (12)
C2	0.0356 (14)	0.0555 (19)	0.0400 (14)	−0.0071 (15)	0.0045 (12)	0.0082 (14)
C3	0.0453 (14)	0.0471 (17)	0.0532 (16)	−0.0149 (15)	−0.0037 (15)	0.0040 (17)
C4	0.0427 (14)	0.0392 (16)	0.0448 (14)	−0.0052 (14)	−0.0053 (13)	−0.0071 (14)
C5	0.0478 (15)	0.0464 (18)	0.0435 (14)	0.0079 (15)	0.0064 (13)	−0.0049 (14)
C6	0.0377 (14)	0.057 (2)	0.0512 (15)	0.0046 (16)	0.0143 (12)	0.0078 (17)
C7	0.0345 (14)	0.0513 (19)	0.0538 (16)	−0.0061 (14)	−0.0005 (13)	0.0095 (17)
C8	0.0348 (13)	0.0461 (16)	0.0416 (13)	−0.0012 (13)	−0.0033 (12)	0.0038 (14)
C9	0.0339 (12)	0.0348 (15)	0.0314 (12)	0.0014 (12)	−0.0027 (11)	0.0011 (12)
C10	0.0434 (15)	0.0391 (16)	0.0358 (14)	0.0024 (13)	−0.0017 (13)	−0.0039 (13)
C11	0.0296 (12)	0.0372 (15)	0.0306 (12)	0.0010 (12)	−0.0026 (11)	0.0048 (12)
C12	0.0317 (12)	0.0346 (14)	0.0342 (12)	0.0011 (13)	−0.0051 (12)	0.0036 (12)
C13	0.0284 (12)	0.0364 (15)	0.0358 (12)	0.0050 (12)	−0.0062 (11)	0.0035 (12)
C14	0.0319 (13)	0.0378 (15)	0.0364 (12)	0.0035 (12)	−0.0001 (12)	0.0014 (13)
N15	0.0347 (11)	0.0497 (14)	0.0418 (11)	−0.0001 (12)	0.0084 (10)	−0.0042 (12)
C16	0.0510 (16)	0.0460 (18)	0.0614 (17)	0.0072 (16)	0.0043 (15)	−0.0056 (16)
C17	0.0407 (14)	0.076 (2)	0.0489 (15)	0.0057 (17)	0.0081 (13)	−0.0116 (16)
O18	0.0404 (10)	0.0629 (12)	0.0418 (10)	−0.0084 (10)	0.0001 (8)	−0.0145 (10)
O19	0.0661 (13)	0.0604 (14)	0.0665 (13)	−0.0108 (11)	0.0160 (11)	−0.0280 (13)

### Geometric parameters (Å, °)

**Table:**

C1—N15	1.373 (3)	C8—H8	0.9300
C1—C2	1.413 (4)	C9—O18	1.230 (3)
C1—C11	1.430 (3)	C9—C11	1.469 (3)
C2—C3	1.360 (4)	C9—C13	1.483 (3)
C2—H2	0.9300	C10—O19	1.227 (3)
C3—C4	1.374 (4)	C10—C14	1.467 (4)
C3—H3	0.9300	C10—C12	1.483 (3)
C4—C12	1.374 (3)	C11—C12	1.420 (3)
C4—H4	0.9300	C13—C14	1.392 (3)
C5—C6	1.370 (4)	N15—C16	1.446 (3)
C5—C14	1.387 (3)	N15—C17	1.459 (3)
C5—H5	0.9300	C16—H16A	0.9600
C6—C7	1.375 (4)	C16—H16B	0.9600
C6—H6	0.9300	C16—H16C	0.9600
C7—C8	1.382 (3)	C17—H17A	0.9600
C7—H7	0.9300	C17—H17B	0.9600
C8—C13	1.389 (3)	C17—H17C	0.9600
			
N15—C1—C2	119.5 (2)	C14—C10—C12	118.5 (2)
N15—C1—C11	122.8 (2)	C12—C11—C1	117.8 (2)
C2—C1—C11	117.7 (2)	C12—C11—C9	117.7 (2)
C3—C2—C1	121.7 (2)	C1—C11—C9	123.7 (2)
C3—C2—H2	119.1	C4—C12—C11	121.4 (2)
C1—C2—H2	119.1	C4—C12—C10	117.5 (2)
C2—C3—C4	120.8 (3)	C11—C12—C10	121.1 (2)
C2—C3—H3	119.6	C8—C13—C14	119.0 (2)
C4—C3—H3	119.6	C8—C13—C9	119.8 (2)
C12—C4—C3	119.9 (2)	C14—C13—C9	121.1 (2)
C12—C4—H4	120.1	C5—C14—C13	119.6 (2)
C3—C4—H4	120.1	C5—C14—C10	120.7 (2)
C6—C5—C14	120.9 (2)	C13—C14—C10	119.7 (2)
C6—C5—H5	119.6	C1—N15—C16	122.26 (19)
C14—C5—H5	119.6	C1—N15—C17	120.3 (2)
C5—C6—C7	119.8 (2)	C16—N15—C17	114.2 (2)
C5—C6—H6	120.1	N15—C16—H16A	109.5
C7—C6—H6	120.1	N15—C16—H16B	109.5
C6—C7—C8	120.2 (2)	H16A—C16—H16B	109.5
C6—C7—H7	119.9	N15—C16—H16C	109.5
C8—C7—H7	119.9	H16A—C16—H16C	109.5
C7—C8—C13	120.5 (2)	H16B—C16—H16C	109.5
C7—C8—H8	119.8	N15—C17—H17A	109.5
C13—C8—H8	119.8	N15—C17—H17B	109.5
O18—C9—C11	122.3 (2)	H17A—C17—H17B	109.5
O18—C9—C13	119.2 (2)	N15—C17—H17C	109.5
C11—C9—C13	118.4 (2)	H17A—C17—H17C	109.5
O19—C10—C14	120.7 (2)	H17B—C17—H17C	109.5
O19—C10—C12	120.8 (2)		
			
N15—C1—C2—C3	−172.0 (2)	O19—C10—C12—C11	−178.1 (2)
C11—C1—C2—C3	6.6 (3)	C14—C10—C12—C11	1.8 (3)
C1—C2—C3—C4	0.2 (4)	C7—C8—C13—C14	−0.9 (3)
C2—C3—C4—C12	−3.7 (4)	C7—C8—C13—C9	−179.9 (2)
C14—C5—C6—C7	−1.0 (4)	O18—C9—C13—C8	14.8 (3)
C5—C6—C7—C8	1.9 (4)	C11—C9—C13—C8	−168.1 (2)
C6—C7—C8—C13	−1.0 (4)	O18—C9—C13—C14	−164.1 (2)
N15—C1—C11—C12	168.8 (2)	C11—C9—C13—C14	12.9 (3)
C2—C1—C11—C12	−9.8 (3)	C6—C5—C14—C13	−0.9 (4)
N15—C1—C11—C9	−21.7 (3)	C6—C5—C14—C10	176.6 (2)
C2—C1—C11—C9	159.7 (2)	C8—C13—C14—C5	1.9 (3)
O18—C9—C11—C12	155.6 (2)	C9—C13—C14—C5	−179.2 (2)
C13—C9—C11—C12	−21.3 (3)	C8—C13—C14—C10	−175.7 (2)
O18—C9—C11—C1	−13.8 (4)	C9—C13—C14—C10	3.3 (3)
C13—C9—C11—C1	169.2 (2)	O19—C10—C14—C5	−8.3 (4)
C3—C4—C12—C11	0.2 (4)	C12—C10—C14—C5	171.8 (2)
C3—C4—C12—C10	−177.5 (2)	O19—C10—C14—C13	169.2 (2)
C1—C11—C12—C4	6.6 (3)	C12—C10—C14—C13	−10.7 (3)
C9—C11—C12—C4	−163.5 (2)	C2—C1—N15—C16	145.7 (2)
C1—C11—C12—C10	−175.8 (2)	C11—C1—N15—C16	−32.9 (3)
C9—C11—C12—C10	14.1 (3)	C2—C1—N15—C17	−12.9 (3)
O19—C10—C12—C4	−0.4 (4)	C11—C1—N15—C17	168.5 (2)
C14—C10—C12—C4	179.5 (2)		

### Hydrogen-bond geometry (Å, °)

**Table:**

*Cg*1 and *Cg*2 are the centroids of the C1–C4/C11/C12 and C5–C8/C13/C14 rings respectively.

**Table:**

*D*—H···*A*	*D*—H	H···*A*	*D*···*A*	*D*—H···*A*
C2—H2···Cg1^i^	0.93	2.99	3.724 (3)	137
C4—H4···Cg2^ii^	0.93	2.81	3.678 (3)	156

Symmetry codes:  (i) −*x*, *y*+3/2, −*z*+3/2; (ii) *x*+3/2, −*y*+1/2, −*z*+1.

### Table 2 π–π interactions (Å,°).

**Table:**

*I*	*J*	*CgI*···*CgJ*	Dihedral angle	*CgI*_Perp	Cg*I*_Offset
1	2^iii^	3.844 (2)	11.13 (12)	3.606 (10)	1.334 (10)
2	1^iv^	3.844 (2)	11.13 (12)	3.606 (10)	1.334 (10)

Symmetry codes: (iii) *x* + 1, *y*, *z*; (iv) *x* – 1, *y* + 1, *z* + 1.Notes: *Cg*1 and *Cg*2 are the centroids of the C1-C4/C11/C12 and C5-C8/C13/C14 rings respectively. Cg*I*···Cg*J* is the distance between ring centroids. The dihedral angle is that between the planes of the rings *I* and *J*. *CgI*_Perp is the perpendicular distance of *CgI* from ring *J*. Cg*I*_Offset is the distance between *CgI* and perpendicular projection of *CgJ* on ring *I*.
